# Integrating digital pathology and mathematical modelling to predict spatial biomarker dynamics in cancer immunotherapy

**DOI:** 10.1038/s41746-022-00636-3

**Published:** 2022-07-12

**Authors:** L. G. Hutchinson, O. Grimm

**Affiliations:** Roche Pharma Research and Early Development, Pharmaceutical Sciences, Roche Innovation Center Basel, F. Hoffmann-La Roche Ltd, Grenzacherstrasse 124, 4070 Basel, Switzerland

**Keywords:** Predictive markers, Predictive medicine

## Abstract

In oncology clinical trials, on-treatment biopsy samples are taken to confirm the mode of action of new molecules, among other reasons. Yet, the time point of sample collection is typically scheduled according to 'Expert Best Guess'. We have developed an approach integrating digital pathology and mathematical modelling to provide clinical teams with quantitative information to support this decision. Using digitised biopsies from an ongoing clinical trial as the input to an agent-based mathematical model, we have quantitatively optimised and validated the model demonstrating that it accurately recapitulates observed biopsy samples. Furthermore, the validated model can be used to predict the dynamics of simulated biopsies, with applications from protocol design for phase 1–2 studies to the conception of combination therapies, to personalised healthcare.

## Introduction

In 2018, 9.6 million people died of cancer despite huge and long-standing efforts to develop a cure^1^. One challenge has been the lack of predictive quantitative methodology that improves clinical trial design, including the scheduling of on-treatment biopsies, identifying the best combination treatments, and patient selection. In particular, during early phase oncology clinical trials one aim is to verify the mode of action of the drug by investigating treatment effects in the tumour tissue by histological methods. To ensure meaningful conclusions regarding the mode of action, the time point at which samples (i.e. tumour biopsies) are taken is critical. Typically a single core-needle biopsy is taken at baseline, and another during the treatment period. Given the ordeal for the patient, the number of biopsy samples taken must be minimised, and the time point needs to be chosen wisely. However, currently, this time point is chosen by clinical experts based on ‘best-guess’ rather than being informed by quantitative scientific approaches. Such approaches can also be used to identify appropriate combination treatment partners as well as to select patients to optimise the likelihood of successful therapy both during clinical trials and routine medical procedures. The impact on and cost for patients, drug developers and healthcare systems are obvious.

Here we describe a quantitative method that integrates digital pathology and mathematical disease modelling to address the issues mentioned above. We have developed a simulation model that is capable of predicting a full-time course of spatial biomarkers, which can be used to identify the most informative time point at which to take an on-treatment biopsy sample in the context of oncology clinical trials.

Spatial biomarkers derived from immunohistochemistry (IHC) images have been shown to contain information that is predictive of prognosis, tumour recurrence or response to treatment via statistical analysis^2–4^, and more recently, machine-learning methods^5,6^. Here, we use a mechanistic modelling approach to utilise the full spatiotemporal resolution of paired biopsy samples to gain insights based on biological hypotheses and to perform predictive simulations.

The underlying algorithm is an agent-based model implemented in Matlab, based on that described by Kather et al.^7^. The model comprises two populations of agents; tumour cells and immune cells laid out on a grid, which behaves according to prescribed rules and ultimately captures emergent spatial dynamics. There are several examples of agent-based models representing tumour-immune interactions which are capable of reproducing realistic spatial features and were recently reviewed^8^. However, the validation of these models has been mostly qualitative, i.e. visual assessment or comparison of simulation results to images of tissue sections at a single time point^7,9,10^. In one example, spatial distributions of cells in simulated samples are quantitatively compared using the radial distribution function^11^. In this work, we use paired clinical samples that were taken at two timepoints for each patient: pretreatment (baseline) and on-treatment, allowing us to validate the dynamic components of the model using the spatial and temporal resolution.

We used data from phase 1 clinical trials for Simlukafusp, an immunomodulatory molecule which localises in the tumour due to its FAP binding, and stimulates the IL2beta/gamma receptors expressed on immune cells. We note that inter-patient variability is significant in such a dataset and that we do not have information on previous treatments or the evolutionary status of individual tumours. However, we assume from^12^ that even the small samples from core-needle biopsies are representative of intra-tumour heterogeneity for a given patient. Due to the lack of a widely accepted comparative measure to compare the spatial distributions of cells in sets of clinical images, we derived a spatial agreement measure (SAM), which we used to optimise and test the model.

To this end, we have simulated the on-treatment immune cell distribution using the baseline biopsies as input and compared the predictions to the observed immune cell distribution from the on-treatment samples, achieving a mean accuracy of 77%. It is striking that a single baseline feature, the spatial distribution of immune cells, is predictive of the on-treatment immune cell distribution with such high accuracy.

Finally, we provide two examples for an application of this method - clinical protocol design (choice of biopsy scheduling) and assessment of potential combination therapy partners.

Box **Integration of digital pathology and mathematical modelling.**

The input data to the model is a spatial map of CD8+T-cells (CD8: a cluster of differentiation 8) which is derived from IHC slides from patient samples. Specifically, using a machine-learning algorithm trained by a human expert to detect cytotoxic CD8+T-cells, IHC stained tissue sections are analysed. This algorithm provides the type (i.e. proliferating or non-proliferating CD8+ cell) and spatial coordinates of each cell in the tumour tissue. This spatial map of CD8 cells is mapped onto a grid and provided to the mathematical model as an input. In mathematical model realisations, each cell on the grid can proliferate, migrate, die or interact with other cells according to probabilistic rules initiated at each time step. The likelihood for a cell to perform an action at any given time step is calculated based on probability distributions and its local neighbourhood. For example, in order for an immune cell to kill a tumour cell, it must be in physical proximity. Thus, spatially resolved input data are key.

## Results

In order to train the model to reproduce biopsy images that are in spatial agreement with observed on-treatment biopsies, we performed a local sensitivity analysis and parameter optimisation. For quantitative comparison between the observed biopsy samples and our model simulations at the corresponding time point, we computed spatial summary statistics and designed an agreement score called the spatial agreement measure (SAM). The model optimisation step revealed the parameter values that performed best according to the SAM. The performance of these parameter sets was validated using a holdout set of patient samples which were not used to train the model. Furthermore, we used the validated model to show that samples taken at around 30 days would provide information on maximum CD8 infiltration at a population level. We also predicted the time course of tissue biomarkers based on two different theoretical immunotherapy combination partners by incorporating their modes of action into the model parameters. Finally, we demonstrate how spatial features vary widely based on initial conditions, i.e. the baseline biopsy. Therefore we propose that the model can also be used in a personalised fashion to predict the optimal time point for biopsy scheduling for individual patients.

### Preprocessing of the images for model input, optimisation and validation

**Fig. 1:**
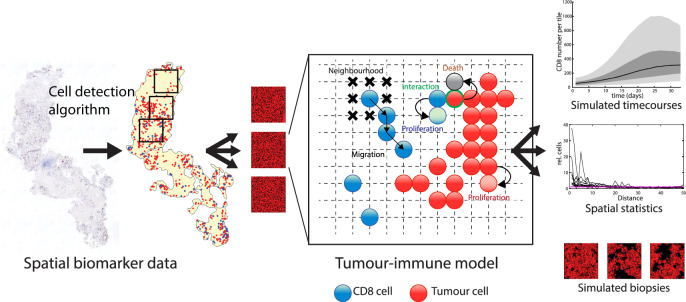
Integrating digital pathology and mathematical modelling. For each patient sample, tissue sections of pre- and on-treatment biopsies were stained against CD8 and Ki67 and digitally analysed to extract the positions of CD8 cells and tumour cells. The image is subdivided into tiles which are used as an input to the model. In the agent-based model, cells at each grid site follow rules regarding their behaviours and interactions. The state of the model is recorded at each time step and the results can be visualised as timecourses and spatial summary statistics and simulated biopsy images.

From the two clinical studies, there were a total of 71 patients from whom paired biopsies were available at the time of data processing. Of these, 44 paired samples included enough tissue in both the pre- and on-treatment biopsy samples to use as an input for the model. These 44 patients were split at random into a training set (*n* = 37) and a test set (*n* = 7). Each pre- and on-treatment biopsy sample was processed using our purpose-built algorithm, and tiles were selected to maximise fields of view and minimise the inclusion of edges and overlaps, as illustrated in Fig. 1. A summary of the training and testing datasets is presented in Tables 1, 2.

**Table 1:** Patient and sample information for patients in the training set and test set.

Patient ID	On-treatment sample time (day since first dose)	N tiles baseline sample	N tiles on-treatment sample	Mean CD8 count cells in baseline tiles	Standard deviation CD8 count in baseline tiles	Mean CD8 count in on-treatment tiles	Standard deviation CD8 count on-treatment tiles
10	63	12	7	19.25	10.98	85.86	34.60
11	49	13	8	84.38	33.13	136.13	26.38
12	49	11	8	293.18	214.96	274.00	148.19
13	49	9	9	46.22	40.67	170.78	95.52
14	113	4	5	60.25	33.39	511.40	217.10
15	48	4	9	146.25	123.17	422.89	99.46
34	57	6	4	157.00	43.25	127.25	7.93
36	48	7	12	85.86	34.60	127.58	49.95
38	62	6	15	219.83	78.20	275.13	97.79
39	57	6	22	239.67	61.29	94.36	38.94
46	49	18	10	165.61	56.71	755.80	153.14
47	57	8	8	9.00	4.57	520.00	149.72
48	50	28	19	139.61	38.11	776.79	147.85
49	50	14	9	68.93	42.00	39.00	38.65
50	50	19	6	63.00	26.93	437.83	130.01
18	42	8	12	221.00	93.15	849.00	307.09
21	43	12	29	23.92	15.44	100.86	106.87
24	26	65	36	51.20	51.58	88.56	54.18
25	17	15	5	312.53	168.81	219.40	86.23
29	15	9	12	22.11	23.96	5.75	9.33
31	24	29	20	60.90	92.49	56.20	50.17
32	17	10	15	124.70	35.39	48.60	67.78
33	15	28	42	118.71	148.46	123.29	153.31
53	49	10	5	1.00	1.70	10.80	8.29
54	57	12	10	20.67	24.96	43.50	34.12
56	42	38	18	59.61	49.76	289.72	170.82
59	43	14	12	70.00	33.56	30.50	17.55
62	52	11	11	59.18	41.43	52.09	29.64
64	48	12	16	124.25	165.33	15.31	9.55
65	44	11	16	163.82	51.69	177.88	81.16
69	16	5	4	17.20	13.55	5.00	5.29
19	49	13	10	50.46	23.34	79.10	91.38
63	42	18	20	5.50	5.11	92.85	102.06
67	30	33	12	64.42	52.15	300.33	95.74
57	44	9	14	101.11	62.76	219.64	102.49
58	45	33	13	8.91	8.97	6.23	9.13
44	50	10	5	11.90	6.21	100.40	43.54
**101**	**23**	**16**	**26**	**62.50**	**43.31**	**167.04**	**113.64**
**102**	**23**	**37**	**25**	**58.46**	**37.46**	**119.12**	**76.81**
**103**	**22**	**8**	**4**	**84.13**	**12.12**	**64.00**	**20.12**
**104**	**23**	**49**	**37**	**180.92**	**114.69**	**196.68**	**93.97**
**105**	**23**	**43**	**9**	**188.70**	**141.22**	**106.11**	**40.42**
**106**	**21**	**7**	**13**	**149.00**	**72.93**	**132.15**	**71.20**
**108**	**23**	**53**	**4**	**22.02**	**33.37**	**106.00**	**57.24**

Bold font rows indicate patients in the test set.

**Table 2:** Indications and biopsy locations are represented in the dataset.

Indication	Training	Test
RCC	16	0
Squamous cell carcinoma	3	5
Adenocarcinoma	1	0
Melanoma skin	2	0
Invasive ductal breast cancer	1	0
Breast adenocarcinoma	4	0
Melanoma	4	0
Adenocarcinoma of oesophagus	1	0
Adenocarcinoma of the cervix	1	0
Adenocarcinoma lung	1	0
Adenocarcinoma of prostate	1	0
Choroid melanoma	1	0
Lung carcinoma	1	0
Invasive breast carcinoma	0	1
Unknown	0	1

Location	Training	Test
Soft tissue	2	0
Lymph node	6	0
Peritoneum	1	0
Kidney	2	0
Stomach	1	0
Lung	5	0
Liver	12	1
Abdominal cavity	1	0
Skin	3	0
Unknown	2	1
Head	1	3
Prostate gland	1	0
Neck	0	2

Using the baseline tiles as the initial condition for model realisations, the model simulates the behaviour of every cell on the grid for a specified period of time-based on rules of proliferation, migration, killing and death. Simulated biopsies are generated and saved at 24 h intervals, creating a rich time course. A schematic that indicates how the data were preprocessed and used as an input to the model simulations and for model optimisation and validation is shown in Fig. 1.

### Derivation of a spatial agreement measure (SAM) based on the radial distribution function

**Fig. 2:**
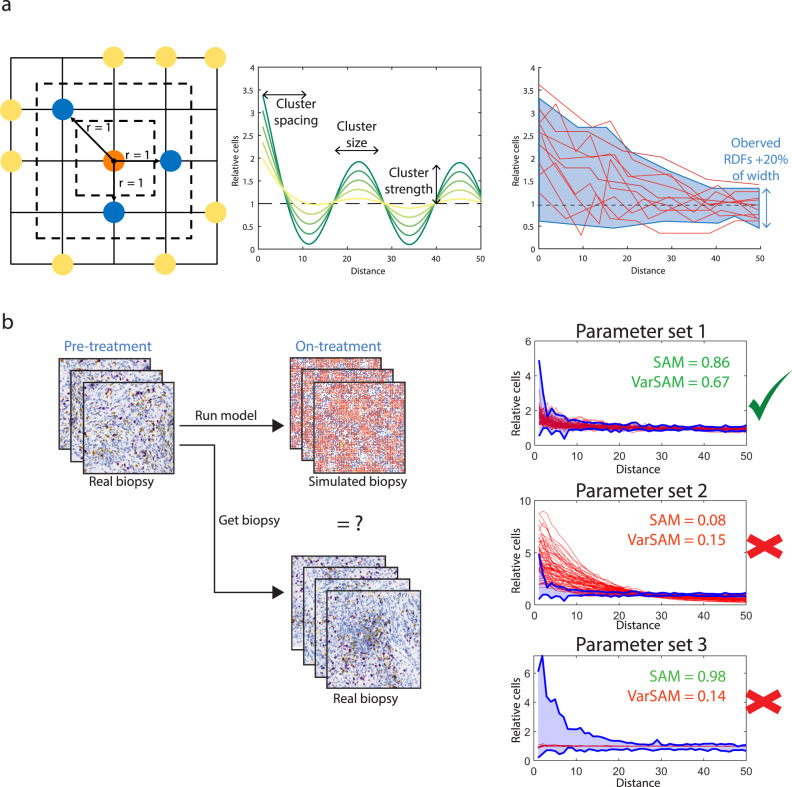
Development of a spatial agreement measure (SAM) for model optimisation and validation. **a** The radial distribution function calculated on a grid is illustrated on the left. From each cell, the Chebyshev distance to every other cell is computed and a histogram is obtained for the total number of cells at each radius. The values are normalised by the mean density of cells on the entire tile. The radial distribution function is plotted as a function of distance as shown in the middle. The characteristics of the radial distribution function correspond to spatial features as indicated. To compare the groups of radial distribution functions on simulated and observed tiles, the SAM is calculated. **b** A conceptual outline of the SAM with examples of accepted and rejected simulations with their respective SAM and VarSAM scores.

The spatial distribution of T-cells is critical for tumour-immune interactions both in biology and in the computational model. Given the stochastic nature of biological events, and indeed the stochastic nature of the computational realisations, an image-to-image comparison must be performed on derived spatial statistics, rather than comparing images directly. Therefore, a comparison of simulated biopsies to real on-treatment biopsies requires a measure of spatial agreement. Since each observed or simulated patient sample is represented by a set of tiles, we wish to compare the spatial distributions of two sets of images to one another. To this end, we developed a method based on the radial distribution function (RDF) as shown in Fig. 2a. The spatial agreement measure (SAM) is a spatial statistic that describes the agreement between two sets of biopsy images, for example, simulated and observed biopsies, by quantifying the overlap of the respective RDF ranges.

In order to capture the variability in the spatial distributions, we provide extra weight to the observed cell density within a short radius of any given CD8 cell, via the VarSAM. The VarSAM is designed to identify artificially high SAM values that result from RDFs that have a narrow distribution. The SAM and VarSAM are summarised pictorially in Fig. 2b.

### Sensitivity analysis: an exploration of model sensitivity

**Fig. 3:**
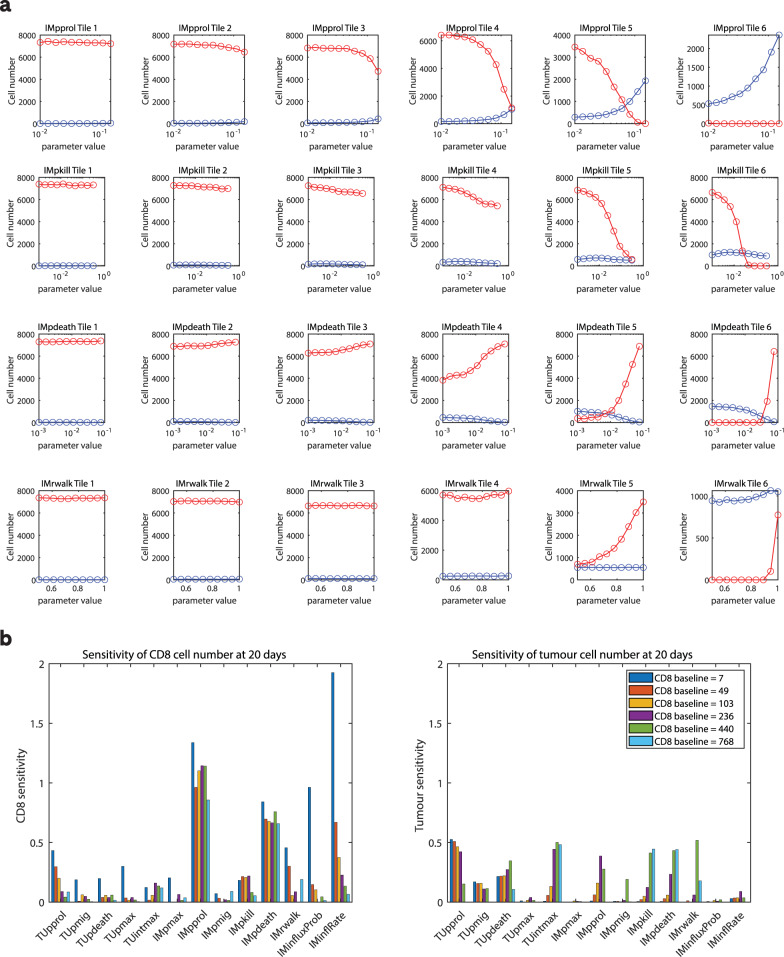
Sensitivity analysis. **a** For six hand-selected tiles reflecting different CD8 numbers and distributions at baseline, and four model parameters, the numbers of CD8 and tumour cells at the 20-day time point are recorded for the range of parameter values scanned. CD8 number is shown in blue and the tumour cell number is shown in red. **b** Summary plot of the sensitivity of 13 model parameters that govern the behaviours of CD8 and tumour cells. The sensitivity was calculated as the change in cell count across the parameter range at day 20 divided by the parameter range scanned. CD8 sensitivity is shown on the left and tumour cell sensitivity is shown on the right.

As shown in Fig. 3, the parameters that displayed the highest sensitivity were those related to the immune cell dynamics, namely the probabilities of CD8 cell proliferation, death, and killing as well as the randomness of CD8 cell migration and the influx rate of immune cells (see Table 4 for the definitions of model parameters). In contrast, several model parameters did not appear to have a strong effect on the numbers of CD8 cells and tumour cells, for example, the proliferation, migration and death rates of tumour cells. Moreover, we note that the number of CD8 cells at baseline influences the sensitivity of the model parameters. It is worth pointing out that our sensitivity analysis is not exhaustive since it is centred on one region of high dimensional parameter space, and therefore does not capture co-dependencies between model parameters. Supplementary Fig. S1 contains further visualisations of the sensitivity analysis results.

### Model optimisation: a set of model parameters can reliably reproduce spatial features of biopsy images

**Fig. 4:**
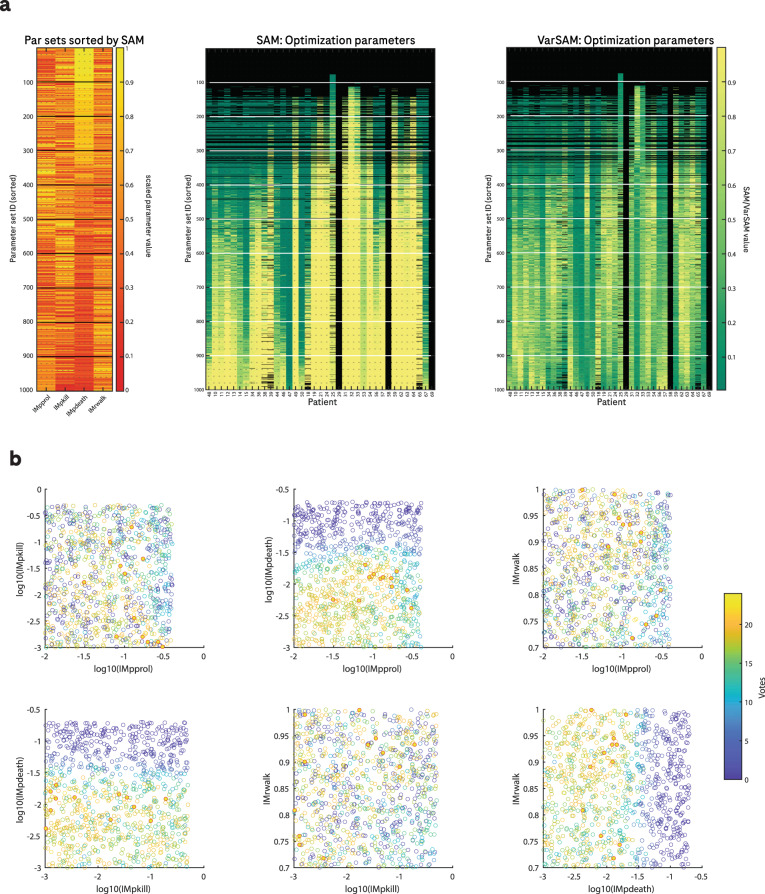
Model optimisation. **a** Heatmaps summarising the optimisation results. Left: normalised parameter values sorted by SAM score. Middle: SAM score for each parameter set for each patient in the training set where green indicates a low SAM (low agreement between simulated and observed samples) and yellow indicates high agreement. Black indicates that there were too few CD8 cells (<10) to make a meaningful evaluation via the SAM. Rows are sorted by the SAM score across all patients. Right: VarSAM values sorted by the SAM score across all patients. Green indicates that simulated biopsies did not capture the variability in the first 15 distance units of the observed RDFs, whereas yellow indicates a good representation of the observed variability. **b** Two-dimensional projections in parameter space of the SAM votes for each sampled parameter set, where one vote corresponds to one patient in the training set for which the SAM was above a threshold of 0.7 and the VarSAM was above a threshold of 0.3. Red circles with a red rim indicate parameter sets that were accepted according to the SAM since they had more than 22 votes.

The model optimisation (summarised in Fig. 4) identified 12 parameter sets that met the criteria for spatial agreement, and these were then used to test the model performance on the holdout dataset and are hereafter referred to as the population parameters. To capture inter-individual variability, we accepted all parameter sets that met the criteria, rather than identifying one top-performing parameter set. We note that the accepted parameters tend to feature a low value for IMpdeath, the probability of spontaneous CD8 cell apoptosis. In contrast, there does not appear to be a clear trend in the values accepted for the other parameters.

The relationships between pairs of parameters and their performance is shown in Fig. 4b. The definitions of model parameters are provided in Table 4. We note that overall there is no noticeable association between accepted parameter values. We also note that a very small proportion of parameter values met our criteria for acceptance (1.2%).

### Null benchmark for predictive accuracy

In order to establish if the agent-based model does indeed add an improvement in the prediction of spatial features compared to predictions in the absence of an underlying model, we wish to determine a reference value for predictive accuracy that the model must exceed in order to be of incremental predictive value. This reference value will be calculated using the last observation carried forward method (LOCF). To this end, we computed the RDFs for the baseline and on-treatment biopsies and calculated the SAM and VarSAM for each patient in the dataset (*n* = 44). Using the same thresholds that were used to accept or reject parameter sets in the model optimisation, we found that for 41% of patients (18 out of 44) the distribution of CD8 cells in the on-treatment sample were well represented by the baseline sample. Therefore in order for the agent-based model to be of predictive value, the final parameter set must produce simulated biopsies that agree with the observed biopsies in more than 41% of cases.

### Model validation: the trained model accurately reproduces spatial biopsy features

**Fig. 5:**
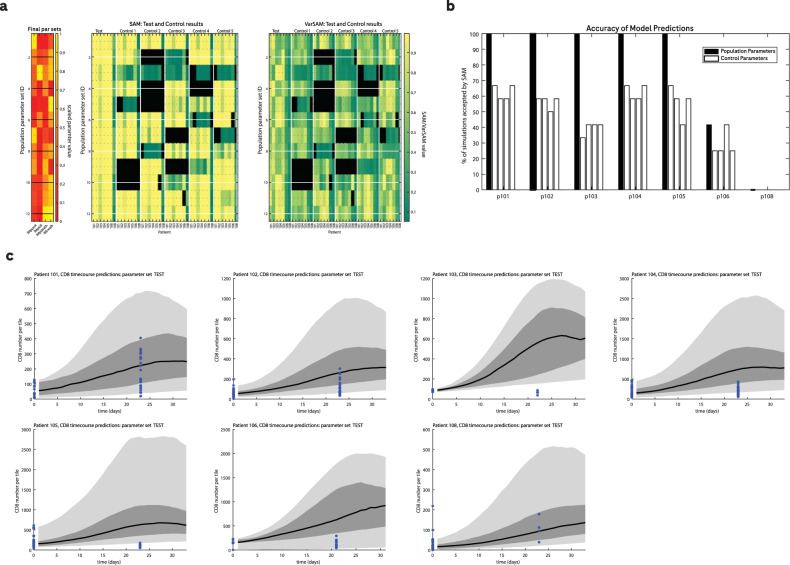
Model validation. **a** Heatmaps showing the SAM and VarSAM of the population parameters applied to the test dataset of seven patients. The first seven columns of the middle and right heatmaps correspond to the simulations using the population parameters and the rest of the columns correspond to simulations using five independent control sets of randomly selected parameter values for comparison. Colours are as in Fig. 4a. **b** Summary bar plot of the heatmaps in figure (**a**). Parameter sets were considered accepted if the SAM had a value of >0.7 and the VarSAM had a value of >0.3. **c** Simulated timecourses of the numbers of CD8 cells using the test parameters with 50% (dark grey) and 90% (light grey) quantiles for each of the patients in the test group. The blue * indicates the actual number of CD8 cells per tile in baseline and on-treatment samples from each patient.

To test the model performance, we ran simulations on the unseen hold-out dataset comprising seven patients using the population parameter values identified in the optimisation step. The results are shown in Fig. 5. Using the SAM to deduce whether simulated biopsies were a faithful representation of the observed on-treatment samples, we found that the population parameter sets were able to reproduce spatial features of on-treatment biopsy samples with a mean accuracy of 77% (and median accuracy of 100%) compared to 35% mean accuracy with randomly selected control parameter sets and the 41% acceptance rate from the LOCF method described above (note that the LOCF was calculated using the full dataset to compensate for the lack of repeats using different parameter sets). For one of the patients in the test set, none of the simulations with the control parameters nor the population parameters were accepted by SAM, which is likely due to the small number of tiles in the on-treatment sample (4) compared to the baseline sample (53). We consider our findings robust to different sample collection times since the on-treatment timepoints for the training set varied between 15–113 days after the first sample was taken. Therefore we are confident that the model is able to reliably predict a full time course of biopsy images including spatial features. It is also noteworthy that a single baseline feature (CD8 spatial distribution) is sufficient model input to predict on-treatment biopsy samples. We note that there is room for improvement for predicting CD8 cell numbers, and this is to be expected since we optimised the model to reproduce the spatial arrangement of CD8 cells rather than CD8 numbers per tile. By performing stochastic repeats and simulations with all accepted parameter sets, we believe we have included sufficient variability to represent inter- and intra-patient variability that arises from, for example, taking biopsy samples from different locations.

### Model application: the model predicts spatial CD8 dynamics for monotherapy and hypothetical combination therapies

An example of the predicted CD8 cell time course for patients in the test set is shown in Fig. 5c. For some patients, the model predicts that the number of CD8 cells levels off or begins to decrease after 30 days, whereas for other patients, the number of CD8 cells continues to increase. Depending on the purpose of biopsy collection, drug development teams may choose to propose a cohort or personalised approach for biopsy collection. Simulations generated with this model can be used to inform the optimal time point for biopsy collection in both cases.

**Fig. 6:**
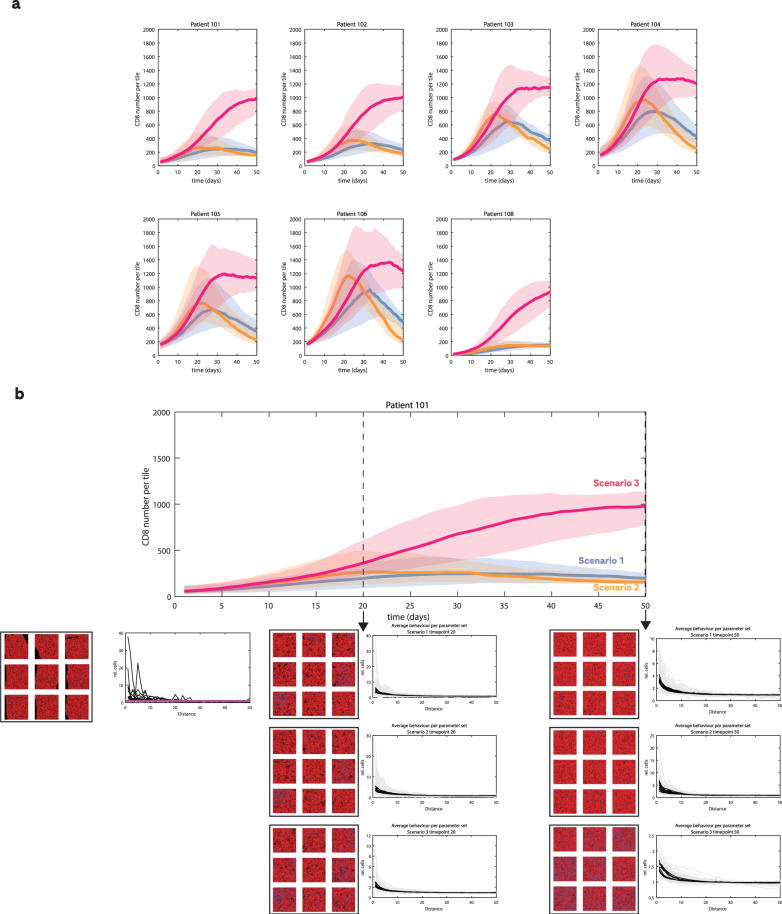
Use of model predictions for clinical trials. **a** Simulations of the CD8 time course for Simlukafusp monotherapy (scenario 1, shown in blue) and two hypothetical combination treatments. The orange line (scenario 2) represents a treatment that would increase the proliferation rate of CD8 cells by two times and the red line (scenario 3) represents a treatment that would increase the influx rate of CD8 cells into the tumour by eight times. Shaded regions correspond to the 50% quantiles from simulations with the population parameters. **b** A full illustration of how the model can be used to inform clinical planning for one example patient from the test set. At each time point, the spatial distribution summary of immune cells, as well as simulated biopsy images, can be extracted, and these can inform drug development teams. In the spatial summary statistics, all simulations are shown in grey and the black lines are the average spatial statistics for each parameter set across all tiles and stochastic repeats. In the simulated biopsy images, red represents tumour cells, blue represents immune cells and black indicates non-tumour tissue.

We present two examples of hypothetical combination therapies as shown in Fig. 6 compared to the monotherapy example which was used to train the model. The first hypothetical combination partner (scenario 2) increases the proliferation probability of CD8 cells and the second (scenario 3) leads to an increase in CD8 influx into the tumour. While both hypothetical drugs lead to an initial increase in CD8 cell number compared to the initial scenario, the molecule that increases the proliferation of CD8 cells, perhaps counterintuitively, leads to a later decrease in CD8 cell number. In contrast, the molecule that increases the influx rate of CD8 cells leads to a sustained increase in CD8 cell number. These emergent phenomena are a major reason to use mathematical and computational models to explore complex biological processes.

In many cases, the spatial distribution of CD8 cells may be a more important biomarker than the total number of CD8 cells. In Fig. 6b we illustrate how simulated biopsies and spatial metrics can also be extracted from the simulations at any chosen time point in order to inform decision-making.

## Discussion

In this article, we have described a predictive scientific approach integrating mathematical modelling and digital pathology. The model has been optimised and tested quantitatively using a novel spatial agreement measure and accurately (mean accuracy 77%) predicts the spatiotemporal distribution of cytotoxic CD8+T cells in tumour tissue. These results encourage us to apply this model to address some of the challenges faced in clinical trials, namely the scheduling of on-treatment biopsies. We view this model as a tool to inform the human decision-maker for biopsy scheduling, perhaps revealing some hidden features that may influence their decision. We assert that such a model may also be used to investigate combination therapy partners or novel targets for immunotherapeutic drugs by exploring parameter regimes which correspond to pharmacological interventions. In addition, we believe that the spatial agreement measure (SAM) we have derived to compare spatial biomarkers of simulated and observed data has broad applications in the fields of digital pathology and mathematical modelling.

Although the mathematical model appears to reliably reproduce the spatiotemporal data on CD8 distribution, naturally it has some limitations. These limitations fall into two classes: clinical operations and model implementation. Given the number of patients typically enroled in early phase clinical trials, the sample size is necessarily small. Furthermore, the size of the tissue sample obtained by needle biopsies is small and often fragmented, which renders some of the datasets unusable. However, for the usable samples, it has been shown that even these small tissue sections usually provide a reliable representation of intra-tumour heterogeneity^12^. Relating to the model implementation, we found that the simulations were heavily influenced by the number of CD8 cells present, and our optimisation process appears to be slightly biased towards choosing parameter sets which lead to an increase in CD8 number. This arises from using a measure to compare the spatial distributions, which is dominated by noise in the case of too few CD8s. In addition, due to computation time and memory restrictions, we were only able to sample 1000 parameter sets for the optimisation with 5 stochastic repeats per simulation. Finally, for the development described in this article, the concentration of the drug in the tumour and its effect on the model parameters was out of scope due to lack of data, however, this is planned for a future version.

Investigations into the dynamic behaviour of complex mechanistic models can reveal both expected and unexpected parameter dependencies, which can be interpreted in terms of their physiological meaning. In the best case, elucidating the effects of physiologically relevant parameters on model outcomes can lead to hypothesis generation for novel modes of action of new drugs. For example, an expected outcome of a sensitivity analysis such as the one performed in this article may be to find that the rate at which CD8 cells kill tumour cells has a strong influence on the numbers of tumour and immune cells. Indeed, there are numerous molecules in development which are intended to increase killing events. An unexpected outcome of our sensitivity analysis was that the randomness of CD8 cell motility also has a notable effect on the model outcome. Mechanistic models such as the one presented here are a valuable tool for investigating novel modes of action for new molecules alone and in combination.

Big data approaches in which labelled data is used to train a machine-learning algorithm are a current trend in personalised healthcare. The advantage of these approaches is, if sufficient data are available, to be able to make a prediction of some future event such as clinical response without mechanistic knowledge of the underlying biological process. On the other hand, using mechanistic modelling one can obtain a full-time course of events (and not just the time point on which the algorithm was trained) and understand the mechanism by which the prediction is made. For example, we have seen that a single feature, the spatial distribution of CD8+T-cells, is sufficient to predict the ground truth with high (77%) accuracy. Machine-learning approaches show a similar performance but the biological or mechanistic interpretation of features is more challenging. The two approaches can be complementary to one another such that a mechanistic model like the one described here generates a full-time course of biomarkers, and the accordingly enriched dataset can then be used to train an ML algorithm.

Regarding the clinical utility of the described approach, a major question during early drug development relates to the mode of action (MoA) of the drug and if indeed it works as it has been designed to do. Researchers investigating or wishing to confirm the MoA of such a new investigational drug could use this model to obtain quantitative guidance on the most informative time point for biopsy collection. For example, the effect of molecules aimed at increasing the number of T-cells in the tumour should be assessed when the assumed effect has resulted in a measurable increase in the latter. To this end, researchers could use CD8 maps from baseline biopsies of the disease of interest and run simulations on different baseline distributions. When additional data becomes available as the clinical trial proceeds, the model can be updated.

The vision we have for drug development is one where tools and approaches such as the one described here are a routine part of all steps in the drug development pipeline guiding human experts in their decisions. As a result, decisions can be reached earlier and more efficiently reducing the cost of drug development and, most importantly, improving patient benefit. Regulators have already begun to appreciate the value of computational approaches and in the future, they may demand quantitative data for decision support.

## Methods

### Data

At the time of data curation, there were 71 patients enroled in two clinical trials for Simlukafusp (NCT03063762 and NCT02627274) who had had baseline and on-treatment biopsy samples. Of these, 44 biopsy pairs contained enough tissue (at least four 100 × 100 cell width fields of view fit within the tissue annotations for baseline and on-treatment samples) to use as an input to the model simulations. In the NCT02627274 trial patients enroled had advanced solid tumours, and in the NCT03063762 trial patients had advanced renal cell carcinoma. Baseline biopsies were usually collected within 7 days before the start of treatment. On-treatment biopsies were obtained at a range of timepoints as described in Table 1.

IHC stained tissue sections (with antibodies against CD8, a marker of T-cells, and Ki67, a marker of proliferation) are digitised and analysed with an in-house developed machine-learning algorithm^13^. First, the colour of images is deconvolved^14^. Next, candidate objects (i.e. parts of the image that might conceivably be a tumour or T cell based on morphological, intensity and colour criteria) are detected and then classified by an expert (i.e. pathologist) trained classifier. To this end, the human expert reviews images where candidate cells have been marked and either accepts or rejects the suggestion. This generated training data is used to train the classifier. In the last step, the performance of the trained algorithm is tested on a hold-out set (unseen images of the same kind). The typical classification accuracy is ~90%. The spatial coordinates (in a whole slide coordinate system) of detected objects, CD8+T cells (proliferating and non-proliferating based on the expression of Ki67) and proliferating tumour cells, is stored in a database. The resulting spatial map of cells from the whole sample is provided as an input to the preprocessing algorithm.

### Technical details

The tumour cell detection algorithm described above only detects tumour cells at a specific stage in their cell cycle (when expressing Ki67). Assuming a typical diameter of a tumour cell to be comparable to that of a CD8 T-cell (~5 µm), we populated empty space within the tumour annotations (which were hand-drawn by a pathologist) with tumour cells up to a density of 70% of the maximum possible with tight packing of cells. The size of each grid cell in our simulations is considered to be one cell width, which we assume is on the order of magnitude of 5 µm. Although alternative grid definitions are possible (such as off-lattice^15^ or hexagonal^16^), in order to be consistent with the original model, we opted to work with a square lattice and single occupancy of grid spaces. We did not observe any artefacts in our simulations due to the lattice choice.

We subdivide each biopsy into as many minimally overlapping square tiles (of dimension 100 × 100 cell widths) as possible. Each of these tiles is passed to the model as an input. The tiling algorithm that we developed selects tiles from the images while obeying the following rules: at least 90% overlap with tissue annotations and tile overlap of ≤10%.

As well as the positions of CD8 and tumour cells in the tiles, the model requires physiological properties of the cells as an input such as proliferation capacity and stem cell-ness. To this end, for each cell at each position, a number of different properties are stored and updated with each iteration of the model. Since there is no way to obtain these properties from the real biopsy images, we assigned them according to realistic estimations. The properties and our allocations at baseline are listed in Table 3.

**Table 3:** Assigned properties of the tumour cells and immune cells in the preprocessing script.

Property	Cells	Distribution	Interpretation
Proliferation capacity	Tumour cells	U(0,10) integer	Tumour cells each have between 0 and 10 proliferation cycles left before death. Proliferating tumour cells were assigned a capacity of 9 and others were assigned per the distribution.
Stem cell-ness	Tumour cells	Assigned with 20% probability	20% of cells are stem cells
Engagement capacity	Tumour cells	U(1,2) integer	Some tumour cells have undergone one T cell interaction event
Engagement status	Tumour cells	0	No tumour cells are currently engaged with a T cell
Proliferation capacity	Immune cells	U(0,8) integer	Immune cells have between 0 and 8 proliferation cycles left before death. Proliferating immune cells were assigned a capacity of 7 and others were assigned per the distribution.
Killing capacity	Immune cells	100	Set to a high value to allow essentially unlimited killing
Engagement status	Immune cells	0	No immune cells are currently engaged with a tumour cell

For each patient in the dataset, the input to the simulation model consists of a stack of tiles extracted from the full pretreatment biopsy image, each of size 100 × 100 cell widths, and a structure containing the properties of the cells in each tile. Depending on the size of the original sample, the number of tiles per patient ranged from 1–65 and the number of tiles per patient are listed in Table 1. We excluded samples from which less than four tiles were extracted to increase statistical confidence in our results.

### Model extension

The mathematical model is based on that from ref. ^7^, which we extended to suit our purpose. Specifically, since we are using tiles from real biopsy samples as an input to the model we replaced the original no-flux boundary conditions with periodic boundary conditions which better reflect the physiological scenario. In addition, we increased the resilience of tumour cells such that several attacks by immune cells were necessary to lead to tumour cell death based on in vivo imaging data^17^. A description of the cell properties assigned for simulations is provided in Table 3 and the model parameters with descriptions are provided in Table 4.

**Table 4:** Parameter names, descriptions and ranges scanned for the sensitivity analysis and optimisation steps.

Parameter	Description	Default value	Values sampled for sensitivity analysis	Values sampled for optimisation
IMpprol	Immune cell proliferation probability	0.049	log scale 0.01–0.15	log scale 0.01–0.4
IMpkill	Immune cell killing probability	0.1	log scale 0.001–0.3	log scale 0.001–0.5
IMpdeath	Natural death probability of immune cells	0.0147	log scale 0.001–0.076	log scale 0.001–0.2
IMrwalk	Randomness of the biased random walk of immune cells	0.8	linear scale 0.5–1	linear scale 0.7–1
TUpprol	Tumour cell proliferation probability	0.5	linear scale 0.05–0.5	na
TUpmig	Tumour cell migration probability	0.35	linear scale 0–0.35	na
TUpdeath	Tumour cell natural death rate	0.12	linear scale 0.01–0.12	na
TUpmax	Tumour cell proliferation capacity	10	linear scale 6–15	na
TUintmax	Tumour cell interaction capacity	2	linear scale 1–10	na
IMpmax	Immune cell proliferation capacity	6	linear scale 3–12	na
IMpmig	Immune cell migration probability	2	linear scale 0.05–0.7	na
IMinfluxProb	Immune cell influx probability	0.2	linear scale 0.01–0.5	na
IMinflRate	Immune cell influx rate	1	linear scale 1–10	na

### Spatial agreement evaluation

In order to evaluate the agreement between real and simulated biopsy samples, we developed a spatial agreement measure (SAM). The spatial distribution of CD8 cells on a given tile is summarised by the normalised RDF, defined in^18^ as the square taxicab paired correlation function. This normalised RDF, which comprises a vector of relative cell densities at each possible inter-cell distance, can be derived from any simulated or observed tile. We assess agreement between collections of tiles from simulated and observed biopsies using these RDF vectors (see Fig. 2a). At each possible inter-cell distance, the relative cell density values in observed and simulated biopsy tiles are compared. Specifically, the proportion of relative cell densities in simulated tiles that fall within the range of relative cell densities from observed tiles ±20% is calculated and compared to a threshold value (*threshSAM*) at each possible inter-cell distance. The calculation is performed for all possible inter-cell distances and the SAM is defined as the proportion of inter-cell distances for which the threshold was met.

Specifically, the SAM is calculated according to the following four steps.

1. Consider the matrices *Ro* and *Rs* such that the rows of *Ro* represent the normalised RDFs for each observed tile of the on-treatment biopsy for a given patient and the rows of *Rs* represent the normalised RDFs for all stochastic repeats for simulated tiles for the same patient at the appropriate time point. The number of rows of *Ro* is the number of tiles identified on the on-treatment biopsy sample and the number of rows of *Rs* is the number of tiles identified on the pretreatment biopsy sample multiplied by the number of stochastic repeats. Both *Ro* and *Rs* have the same number of columns, which is defined by the length of the RDF, *M*, in this case, half of the computational domain length, equal to 50 distance units. To summarise *Ro*_*ij*_ is the normalised paired correlation function at distance *j* for the *i*th tile from the observed on-treatment biopsy and *Rs*_*kj*_ is the normalised paired correlation function at distance *j* for the *k*th simulation of the baseline biopsy.
2. Identify the upper (*u*) and lower (*l*) threshold vectors for RDF acceptance from the RDFs of observed tiles *Ro* such that
  $$u_j = \mathop {{\max }}\limits_i Ro_j + 0.2 \times \mathop {{{{{\mathrm{range}}}}}}\limits_i \;Ro_j,$$
  $$l_j = \max \left( {\mathop {{\min }}\limits_i Ro_j - 0.2 \times \mathop {{{{{\mathrm{range}}}}}}\limits_i Ro_j,0} \right).$$
3. For each RDF distance *j*, find the number of RDFs from simulated tiles *Rs* that lie within the bounds defined by *u* and *l* and divide by the total number of simulations, *N*, to obtain the vector of a proportion of accepted simulated RDFs for each distance, *A*:
  $$A_j = \frac{{\mathop {\sum }\nolimits_k \left( {l_j \,<\, Rs_{kj} \,<\, u_j} \right)}}{N}.$$
4. Find the number of RDF distances for which the proportion of simulated RDFs that fall within the accepted range is at least *threshSAM* and divide by the maximum distance *M* to obtain the SAM.
  $${SAM} = \frac{{\mathop {\sum }\nolimits_j A > {threshSAM}}}{M}.$$

The distribution of CD8 cells in the immediate vicinity provides important information regarding the size and frequency of CD8 clusters^18^. To ensure that the vicinity is given appropriate weight, we perform a second SAM step to quantify the agreement between the ranges of RDF values within the first 15 distance units. The VarSAM is the ratio of the ranges of the RDFs of simulated and observed biopsies in the first 15 distance units. Figure 2a summarises the SAM and VarSAM.

Mathematically, the VarSAM is defined as

$${{VarSAM}} = \min \left( {\frac{{{{range}}\left( {Rs\left( {i\, <\, 16} \right)} \right)}}{{{{range}}\left( {Ro\left( {i \,< \,16} \right)} \right)}},\frac{{{{range}}\left( {Ro\left( {i \,<\, 16} \right)} \right)}}{{{{range}}\left( {Rs\left( {i \,< \,16} \right)} \right)}}} \right).$$

### Sensitivity analysis methodology

For the model sensitivity analysis, simulations were performed using parameter values within realistic physiological ranges, sampled either linearly or logarithmically as summarised in Table 4. In light of the computing power available, the parameters were varied independently. The simulations were performed for a hand-selected set of six tiles from different patients which had different numbers of CD8 cells at baseline, covering a range between 7 and almost 800 CD8 cells per tile. Every simulation was repeated five times with different random seeds, and an average of the simulation results was calculated. The sensitivity of CD8 cell number and tumour cell number to changes in the parameter values was calculated at the 20-day time point of the simulations according to

$${\mathrm{CD8}}\;{\mathrm{sensitivity}} = \frac{{({{CD8}}({{max}}) - {{CD8}}({{min}}))/{{CD8}}({{baseline}})}}{{({{par}}({{max}}) - {\mathrm{par}}({{min}}))/{{par}}({{mid}})}}$$

such that par(max) and par(min) are the maximum and minimum values of the explored parameter range and CD8(max) and CD8(min) CD8 cell number are the corresponding CD8 cell number for simulations using those parameter values. The CD8 cell number is normalised by the number of CD8 cells at baseline and the parameter range is normalised by the midpoint of the range scanned (par(mid)). An analogous expression was used to calculate tumour cell sensitivity.

Longitudinal plots of cell numbers against time for the sensitivity analysis simulations are provided in Supplementary Fig. 1.

### Optimisation methodology

For the model optimisation, we selected 1000 sets of the four chosen parameters (IMpprol, IMpdeath, IMrwalk and IMpkill) from uniform or logarithmic distributions within realistic physiological ranges, as indicated in Table 4. Model simulations were performed using each parameter set for every tile, for every patient in the training set, and the simulations were repeated five times to allow averaging over stochastic runs. The end-time of the simulation was set according to the real time-point of the on-treatment biopsy sample for each patient. The spatial distribution of CD8 cells from observed biopsies was compared with that of the simulated biopsies via the SAM, providing a score between 0–1 for every parameter set, for every patient as summarised in the heatmap in Fig. 4. There are several circumstances in which there are exceptions to this method which are handled as follows:

1. In cases where the simulations ended before the prescribed end-time (usually due to the tumour being eradicated), the last simulated time point was used to calculate the SAM.
2. In cases where there are very few CD8 cells on either the simulated or observed biopsy sample, the RDFs are dominated by noise and the SAM is no longer considered representative.
  - Whenever the majority of tiles from the observed on-treatment biopsy for any given patient contained less than ten CD8 cells, we excluded these samples from the SAM calculations.
  - In the case that there were less than 10 CD8 cells in the majority of simulated tiles for any given parameter set, the SAM was not calculated and a NaN was recorded. In these cases, rather than comparing the spatial distribution of CD8 cells in collections of tiles, the number of CD8 cells per tile is compared instead. Specifically, the two-sample *t*-test was calculated to evaluate whether the total CD8 cells per tile in observed and simulated tiles could belong to the same underlying distribution, and therefore whether the parameter set should be accepted.

In order to identify parameter sets that generated realistic on-treatment biopsies across the population, we ranked the parameter sets by the mean SAM across all patients and accepted all parameter sets where the SAM was >0.7. From the selected parameter sets, we excluded those that would not yield sufficient variability as measured by the VarSAM (explained above). The threshold for VarSAM was 0.3. The resulting final population parameter set contains 12 parameter combinations that we consider to produce the most realistic biological behaviour.

#### Model validation

For the model validation, we used the 12 population parameters identified through the model optimisation, along with the baseline CD8 distributions for the unseen holdout dataset of seven patients to perform simulations (with five stochastic repeats per parameter set). As a control, we repeated this exercise five times with 12 randomly chosen parameter sets. To compare the simulated spatial distributions of CD8 cells with the observed distributions of CD8 cells for the test patients, we calculated the SAM as described in the previous section. We consider the model accuracy to be defined as the percentage of accepted simulations according to the same thresholds that were used for the optimisation, (SAM > 0.7 and VarSAM > 0.3).

#### Ethics statement

All participants provided written informed consent to take part in the study and the study was approved by the ethics committee of Hoffman-La Roche.

### Reporting summary

Further information on research design is available in the Nature Research Reporting Summary linked to this article.

## Supplementary information

Supplemental Material

Reporting Summary

## Data Availability

The cell position (gridstack) data is available as a zipped file at the following location 10.5281/zenodo.6631677. For up-to-date details on Roche’s Global Policy on the Sharing of Clinical Information and how to request access to related clinical study documents, see here: https://go.roche.com/data_sharing.

## Code availability

All code is located at the following public repository 10.5281/zenodo.6631666.
