# Supplementary material for: Integrating digital pathology and mathematical modelling to predict spatial biomarker dynamics in cancer immunotherapy
Source: NPJ Digit Med. 2022 Jul 12;5:92. doi: 10.1038/s41746-022-00636-3 (PMC9276679; doi:10.1038/s41746-022-00636-3)
Supplement: Supplementary file 1 — Supplemental Material [file 41746_2022_636_MOESM1_ESM.pdf]

Sensitivity analysis: median cell number over time (1/4)

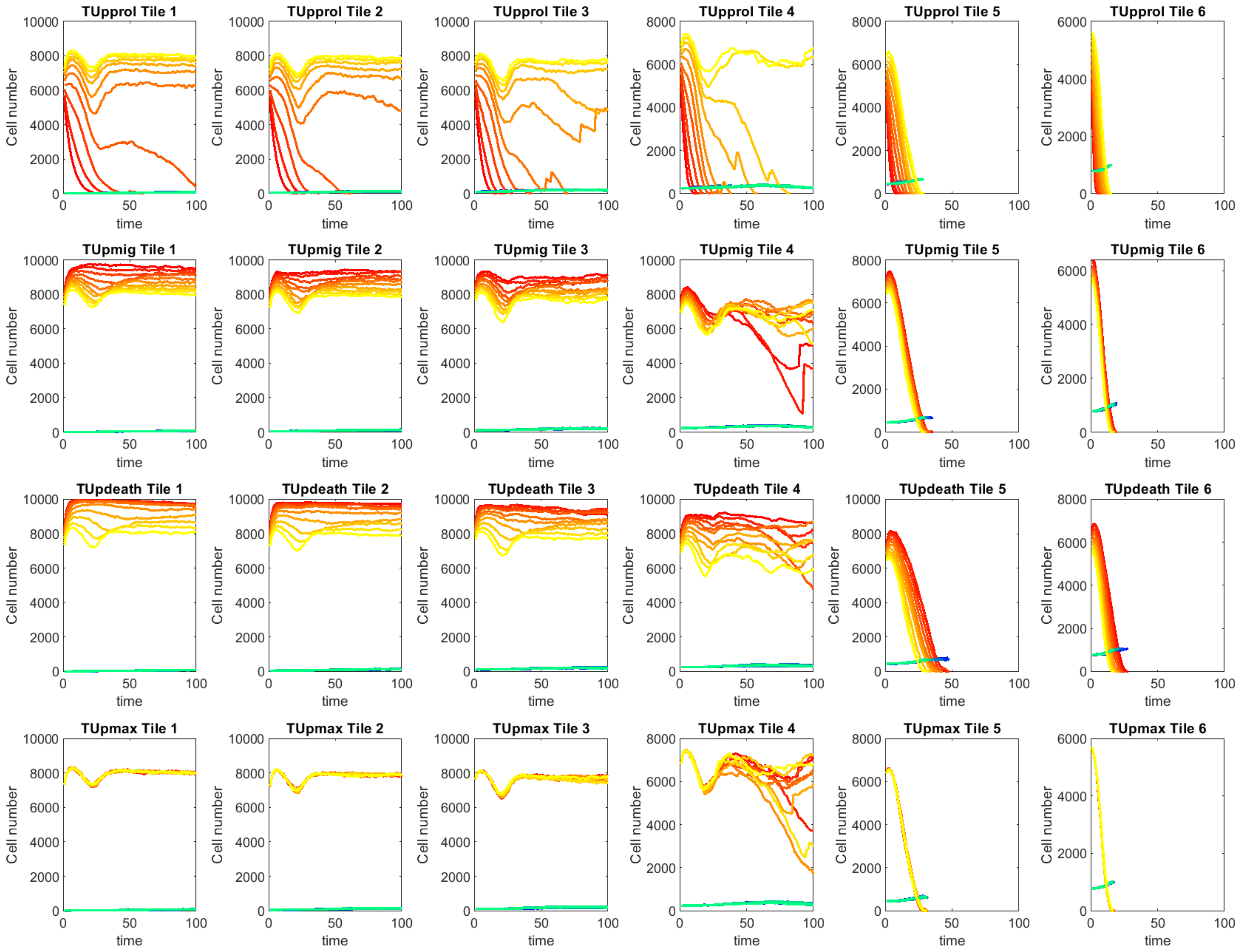

Sensitivity analysis: median cell number over time (2/4)

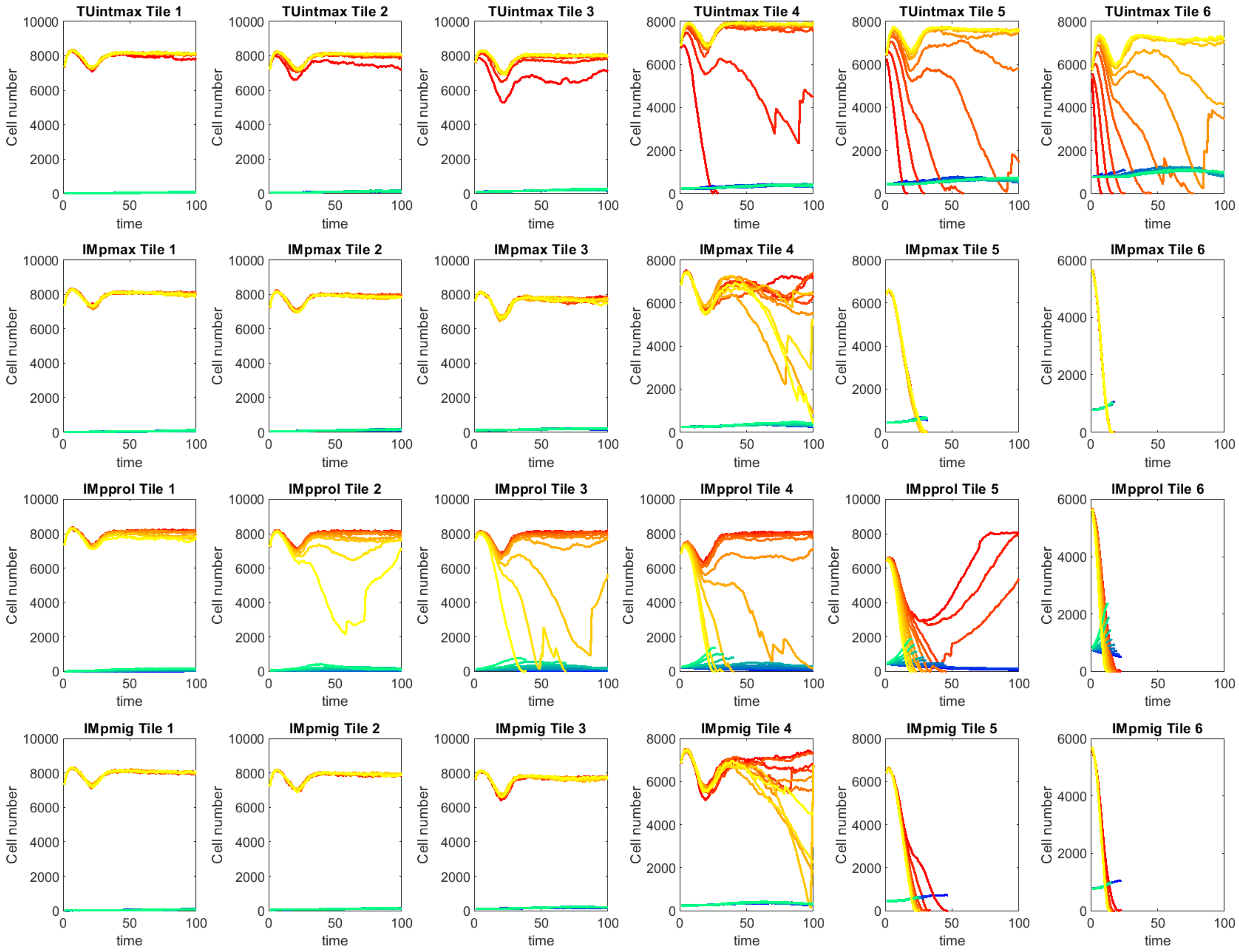

Sensitivity analysis: median cell number over time (3/4)

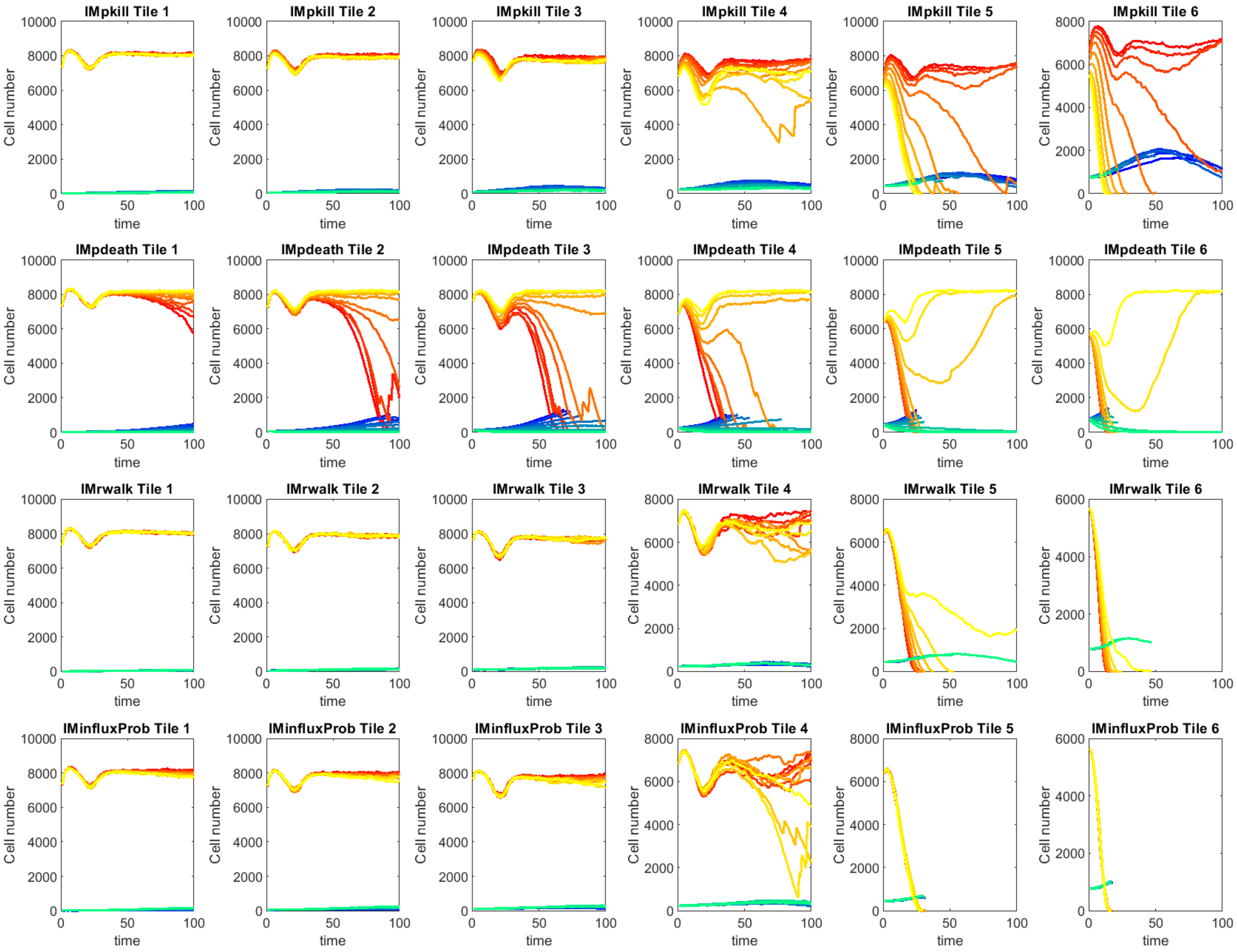

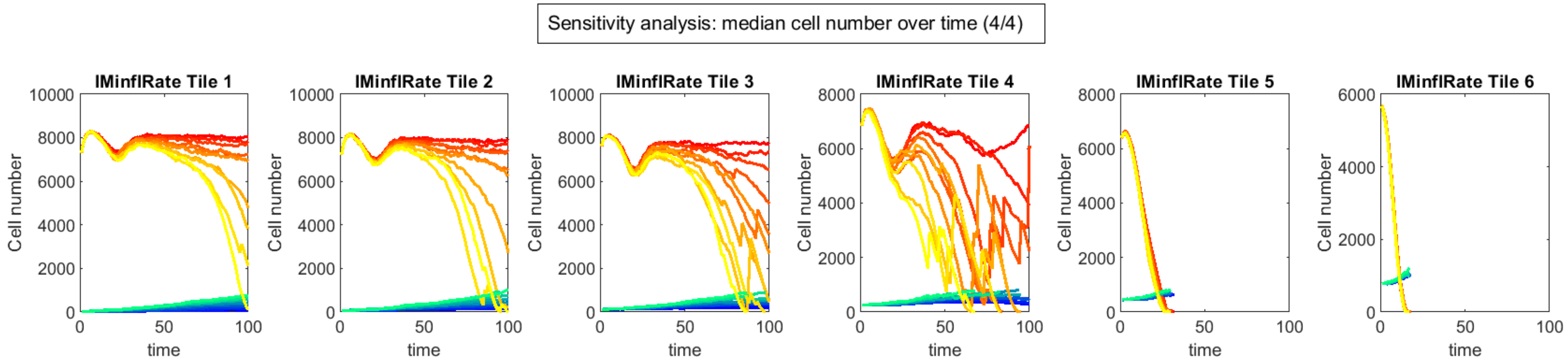

Supplementary Figure 1. Sensitivity analysis: Longitudinal plots to show the median total cell number per tile for 6 selected tiles for simulations of 100 days. Line colour represents parameter values and the blue-green lines represent CD8 cell number (blue=lowest parameter value and green=highest parameter value) while the red-yellow lines represent tumour cell numbers (red=lowest parameter value and yellow=highest parameter value).

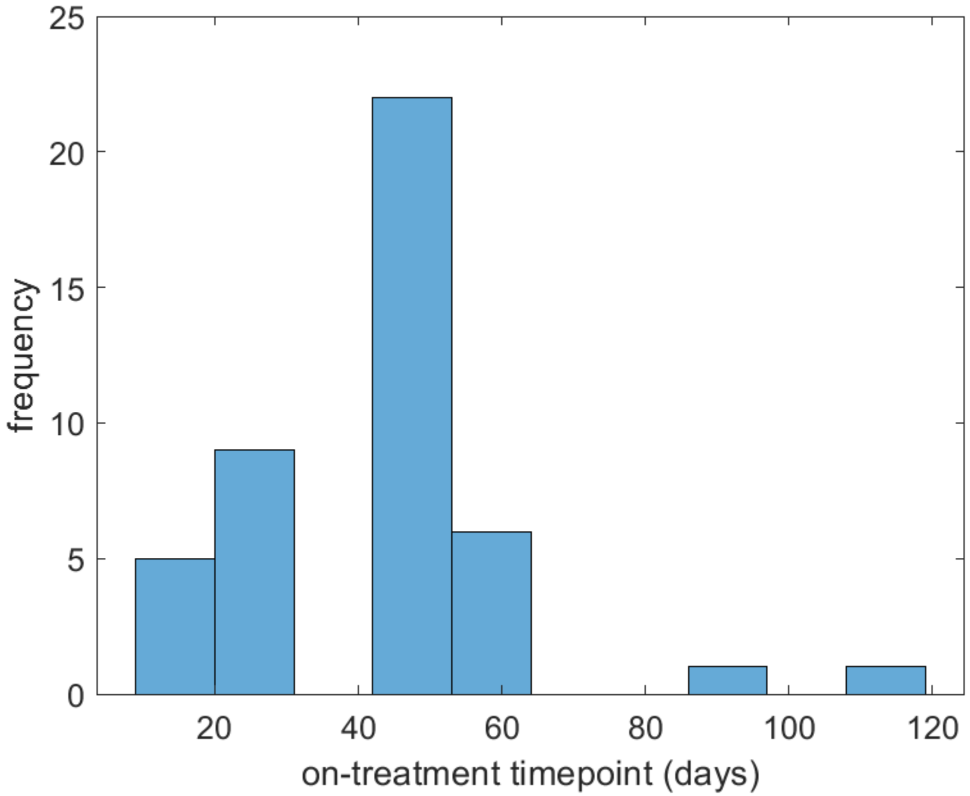

Supplementary Figure 2. A histogram to show the time points at which on-treatment biopsy samples were taken for all patients in the dataset (n=44).
