# Supplementary material for: Integrating digital pathology and mathematical modelling to predict spatial biomarker dynamics in cancer immunotherapy
Source: NPJ Digit Med. 2022 Jul 12;5:92. doi: 10.1038/s41746-022-00636-3 (PMC9276679; doi:10.1038/s41746-022-00636-3)
Supplement: Supplementary file 2 — Reporting Summary [file 41746_2022_636_MOESM2_ESM.pdf]

nature portfolio

Corresponding author(s): Lucy G Hutchinson
Last updated by author(s): May 9, 2022

Reporting Summary

Nature Portfolio wishes to improve the reproducibility of the work that we publish. This form provides structure for consistency and transparency in reporting. For further information on Nature Portfolio policies, see our Editorial Policies and the Editorial Policy Checklist.

Statistics

For all statistical analyses, confirm that the following items are present in the figure legend, table legend, main text, or Methods section.

n/a	Confirmed	
☐	☒	The exact sample size (n) for each experimental group/condition, given as a discrete number and unit of measurement
☒	☐	A statement on whether measurements were taken from distinct samples or whether the same sample was measured repeatedly
☐	☒	The statistical test(s) used AND whether they are one- or two-sided<br>*Only common tests should be described solely by name; describe more complex techniques in the Methods section.*
☐	☒	A description of all covariates tested
☒	☐	A description of any assumptions or corrections, such as tests of normality and adjustment for multiple comparisons
☐	☒	A full description of the statistical parameters including central tendency (e.g. means) or other basic estimates (e.g. regression coefficient) AND variation (e.g. standard deviation) or associated estimates of uncertainty (e.g. confidence intervals)
☒	☐	For null hypothesis testing, the test statistic (e.g. F, t, r) with confidence intervals, effect sizes, degrees of freedom and P value noted<br>*Give P values as exact values whenever suitable.*
☒	☐	For Bayesian analysis, information on the choice of priors and Markov chain Monte Carlo settings
☒	☐	For hierarchical and complex designs, identification of the appropriate level for tests and full reporting of outcomes
☒	☐	Estimates of effect sizes (e.g. Cohen's d, Pearson's r), indicating how they were calculated

Our web collection on statistics for biologists contains articles on many of the points above.

Software and code

Policy information about availability of computer code

Data collection	We have used custom written Matlab code (included in the submission) to prepare all data used in the manuscript.
Data analysis	We have used custom written Matlab code (included in the submission) to analyze any data in the manuscript.

For manuscripts utilizing custom algorithms or software that are central to the research but not yet described in published literature, software must be made available to editors and reviewers. We strongly encourage code deposition in a community repository (e.g. GitHub). See the Nature Portfolio guidelines for submitting code & software for further information.

Data

Policy information about availability of data

All manuscripts must include a data availability statement. This statement should provide the following information, where applicable:

- Accession codes, unique identifiers, or web links for publicly available datasets
- A description of any restrictions on data availability
- For clinical datasets or third party data, please ensure that the statement adheres to our policy

All data needed to reproduce the results in the paper have been made available as a zipped file.

Field-specific reporting

Please select the one below that is the best fit for your research. If you are not sure, read the appropriate sections before making your selection.

☒ Life sciences ☐ Behavioural & social sciences ☐ Ecological, evolutionary & environmental sciences

For a reference copy of the document with all sections, see nature.com/documents/nr-reporting-summary-flat.pdf

Life sciences study design

All studies must disclose on these points even when the disclosure is negative.

Sample size	We have used all data available in the simlukasfusp clinical trials (NCT03063762 and NCT02627274) and for which the biopsy was of sufficient size.
Data exclusions	We excluded those patients where the biopsy was too small to be of statistical value.
Replication	Given used several (n=5) stochastic repeats to replicate the simulations.
Randomization	We randomly (via the random number generator in matlab) selected the test group among all available data.
Blinding	At the time of the simulations we did not know which patients belonged to training or test group.

Reporting for specific materials, systems and methods

We require information from authors about some types of materials, experimental systems and methods used in many studies. Here, indicate whether each material, system or method listed is relevant to your study. If you are not sure if a list item applies to your research, read the appropriate section before selecting a response.

Materials & experimental systems

n/a	Involved in the study
☒	☐ Antibodies
☒	☐ Eukaryotic cell lines
☒	☐ Palaeontology and archaeology
☒	☐ Animals and other organisms
☐	☒ Human research participants
☐	☒ Clinical data
☒	☐ Dual use research of concern

Methods

n/a	Involved in the study
☒	☐ ChIP-seq
☒	☐ Flow cytometry
☒	☐ MRI-based neuroimaging

Human research participants

Policy information about studies involving human research participants

Population characteristics	Indications and biopsy locations are described in table 2.
Recruitment	We used all data from the simlukasfusp clinical trials (NCT03063762 and NCT02627274).
Ethics oversight	Ethics committee of Hoffman-La Roche

Note that full information on the approval of the study protocol must also be provided in the manuscript.

Clinical data

Policy information about clinical studies

All manuscripts should comply with the ICMJE guidelines for publication of clinical research and a completed CONSORT checklist must be included with all submissions.

Clinical trial registration	NCT03063762 and NCT02627274
Study protocol	https://clinicaltrials.gov/ct2/show/NCT03063762 and https://clinicaltrials.gov/ct2/show/NCT02627274
Data collection	https://clinicaltrials.gov/ct2/show/NCT03063762 and https://clinicaltrials.gov/ct2/show/NCT02627274
Outcomes	In our research the outcome is a simulated biopsy which we compared to the observed on-treatment biopsy via a spatial agreement measure.
